# Pax6 Represses Androgen Receptor-Mediated Transactivation by Inhibiting Recruitment of the Coactivator SPBP

**DOI:** 10.1371/journal.pone.0024659

**Published:** 2011-09-15

**Authors:** Julianne Elvenes, Ernst Ivan Simon Thomassen, Sylvia Sagen Johnsen, Katrine Kaino, Eva Sjøttem, Terje Johansen

**Affiliations:** Molecular Cancer Research Group, Institute of Medical Biology, University of Tromsø, Tromsø, Norway; University of Hong Kong, Hong Kong

## Abstract

The androgen receptor (AR) has a central role in development and maintenance of the male reproductive system and in the etiology of prostate cancer. The transcription factor Pax6 has recently been reported to act as a repressor of AR and to be hypermethylated in prostate cancer cells. SPBP is a transcriptional regulator that previously has been shown to enhance the activity of Pax6. In this study we have identified SPBP to act as a transcriptional coactivator of AR. We also show that Pax6 inhibits SPBP-mediated enhancement of AR activity on the AR target gene probasin promoter, a repression that was partly reversed by increased expression of SPBP. Enhanced expression of Pax6 reduced the amount of SPBP associated with the probasin promoter when assayed by ChIP in HeLa cells. We mapped the interaction between both AR and SPBP, and AR and Pax6 to the DNA-binding domains of the involved proteins. Further binding studies revealed that Pax6 and SPBP compete for binding to AR. These results suggest that Pax6 represses AR activity by displacing and/or inhibiting recruitment of coactivators to AR target promoters. Understanding the mechanism for inhibition of AR coactivators can give rise to molecular targeted drugs for treatment of prostate cancer.

## Introduction

The androgen receptor (AR) and androgens have a central role in development and maintenance of the male reproductive system and in the etiology of prostate cancer [1], [2], the most commonly diagnosed invasive cancer in men in the USA and other western countries [3]. Since prostate cancers are dependent on androgens for development, growth and survival, inhibition of AR activity is a major therapeutic goal for management of the disease (reviewed in [4]). Unliganded AR is mainly localized to the cytoplasm through interactions between its ligand binding domain (LBD) and heat-shock proteins (HSPs). Binding of androgens to the LBD induces conformational changes that cause dissociation of the HSPs [2]. The receptor subsequently dimerizes and becomes phosphorylated, before the receptor-steroid complex is imported into the nucleus. Here it binds with high affinity to specific DNA-regulatory elements of target genes [1]. The context of the promoter and the relative levels of coactivators and corepressors determine the resulting transcriptional outcome of the DNA bound receptor [5]. AR has four functional domains; the divergent N-terminal domain (NTD), a central DNA-binding domain (DBD), a hinge-region, and a C-terminal LBD [1], [2]. The NTD contains poly-glutamine and -glycine repeats as well as the transcriptional activation function-1 site (AF-1) that is constitutively active independent of androgens [6]. The LBD has a second activator function site (AF-2) that is only active in the presence of androgens [2]. Both AF-1 and AF-2 can activate AR, whereas interactions between them can modulate its activity [7].

Stromelysin-1 PDGF-responsive element Binding Protein (SPBP) is a 220 kDa nuclear protein displaying structural and functional properties of a transcriptional regulator. It contains an N-terminal TAD, three nuclear localization signals (NLS), a DBD containing an AT-hook motif, and a C-terminal extended plant homeodomain (ePHD) [8]. SPBP enhances the activity of the transcription factors c-Jun, Pax6, Sp1 and Ets1 [8], [9], and knockdown of *SPBP* significantly reduces the expression of the Ets1 target gene *MMP3* in cells [10]. SPBP has also been identified as an interaction partner for activated Estrogen Receptor α (ERα), and found to work as a corepressor of this particular steroid hormone [11]. Further, SPBP is shown to bind and enhance the activation potential of the transcriptional cofactor RNF4 [12]. RNF4 was originally identified as a coactivator of AR, enhancing both basal and ligand-induced AR-mediated transcriptional activity [13].

Pax6 is an evolutionary conserved transcription factor consisting of two DBDs, the paired domain (PD) and the paired-type homeodomain (HD), separated by a glycine-rich linker sequence [14]–[16]. In addition, a TAD is located in the C-terminal region [17]. The N-terminal PD is structurally and functionally divided into the PAI and RED subdomains [18], [19]. These subdomains, as well as the HD, adopt helix-turn-helix motifs that can bind independently to DNA [19]. The PD can also bind to the HD of Pax6, and to other HD containing proteins [20], [21]. Pax6 is important for embryogenesis of the eye, brain, spinal cord, pancreas, and olfactory epithelium [14], [16], [22]. The developmental role of Pax6 and the other Pax family proteins has been established for quite some time. More recently, it has been acknowledged that *PAX* genes are expressed in cancer cells and tissues where they are not normally found [23]. While most Pax family members are associated with an unfavorable outcome of the cancer, Pax6 has been proposed to have a tumor suppressor function in both glioblastoma [24]–[28] and prostate cancer [29].

In this study, we have identified SPBP as a transcriptional coactivator of AR. SPBP interacts with the DBD of AR via its own DBD. We also mapped the interaction between AR and the AR repressor and developmental transcription factor Pax6 to involve their respective DBDs. Importantly, Pax6 inhibited the SPBP-mediated enhancement of AR activity on the AR targeted probasin promoter. This repression was partly reversed by increased expression of SPBP. Chromatin immunoprecipitations showed that over-expression of Pax6 resulted in less SPBP associated with the probasin promoter. *In vitro* binding studies revealed that Pax6 and SPBP compete for binding to the AR(DBD). This suggests that Pax6 represses AR activity by displacing and/or inhibiting recruitment of coactivators to AR target promoters.

## Materials and Methods

### Plasmid constructs

Plasmids and primers used in this study are listed in Tables S1 and S2, respectively. Details on their construction are available on request. Primers for PCR and DNA sequencing were purchased from Eurogentec and Operon. PCR amplification of cDNA was done using either Pfu Turbo (Stratagene) or ExTaq (TaKaRa) polymerases. Many of the plasmids were made using the Gateway cloning system (Invitrogen). All constructs were verified by DNA sequencing with BigDye v3.1 (Applied Biosystems).

### Cell lines

HeLa (ATCC CCL2), Du145, and B3 (ATCC CRL-11421) cells were grown in Eagle's minimum essential medium (MEM). HEK293 (ATCC CRL-1573), SKNBe(2), and MEF cells were grown in Dulbecco's modified Eagle's medium (DME). PC3 cells were grown in Ham's F12K supplemented with 1.5 g/l sodium bicarbonate, while Kelly and LNCaP cells were grown in RPMI 1640 medium. The medium for the LNCaP cells were supplemented with 1.5 g/l sodium bicarbonate, 4.5 g/l glucose, 10 mM HEPES, and 0.1 mM sodium pyruvate. The GFP-AR 3108 cell line [30] was maintained in DME supplemented with 1 mg/ml G418, 0.55 mg/ml puromycin and 10 mg/ml tetracycline. Stable 3×Flag-Pax6 transfected HeLa cells were generated using the FlpIn recombinant system (Invitrogen) according to the manufacturer's protocol. The HeLa FlpIn 3×Flag-Pax6 cells were grown in DME with 4 µg/ml blasticidin and 200 µg/ml hygromycin. In addition, the media for all cell lines were supplemented with 10% fetal bovine serum (Biochrom AG, S0615) and 1% streptomycin-penicillin (Sigma, P4333). Cultured cells were maintained at 37°C with 95% air and 5% CO_2_ in a humidified atmosphere.

### Reporter gene assays

**Figure 1:**
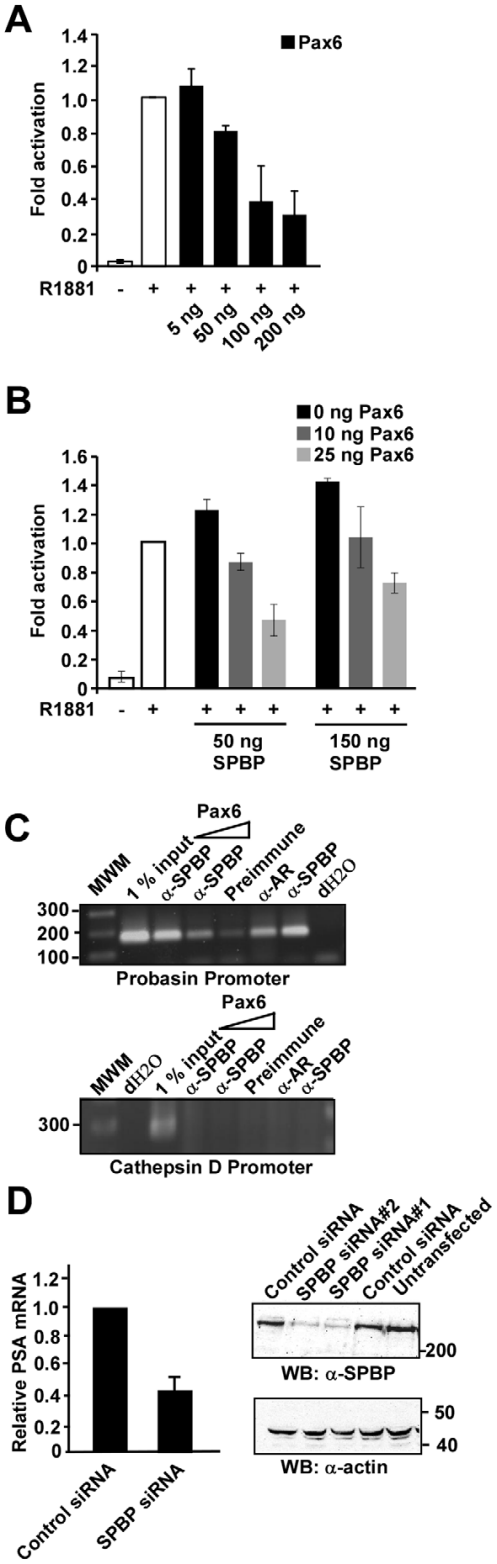
Pax6 represses SPBP-mediated enhancement of AR activity. (A) Pax6 represses AR-mediated expression from the 285PB-Luc reporter. LNCaP cells were cultured in medium with charcoal treated serum and cotransfected with 5–200 ng pDestHA-Pax6, 75 ng pDestHA-AR, 75 ng p285PB-Luc, and 5 ng pCMV-βgal using Metafectene Pro (Biontex). pcDNA3HA was used to equalize the amount of DNA in each well. 10^−7^ M synthetic androgen (R1881) was added as indicated. The mean luciferase/β galactosidase value of AR-mediated expression from 285PB-Luc after stimulation with R1881 was set to 1. The data is based on the mean values of three independent experiments performed in triplicate. (B) SPBP enhances AR-mediated expression, a coactivation that is lost by coexpression of Pax6. HEK293 cells were cultured in medium with charcoal treated serum and cotransfected with 75 ng pDestHA-AR, 50 or 150 ng pDestHA-SPBP, 0–25 ng pDestHA-Pax6, 75 ng p285PB-Luc, and 5 ng pCMV-βgal. The experiment was performed and data obtained as described in A. (C) Chromatin Immunoprecipitation assays show that both SPBP and AR associate with the probasin promoter, and that overexpression of Pax6 decreases the amount of SPBP associated with the promoter. Extracts from HeLa cells cotransfected with 285PB-Luc, pDestHA-SPBP, pSG5-AR, and/or increasing amounts of pDestHA-Pax6 were immunoprecipitated with preimmune serum, polyclonal anti-SPBP antibody or polyclonal anti-AR antibody. PCR analyses on the immunoprecipitated chromatin were carried out using primers flanking the probasin promoter (upper panel). Primers aligning to position -3351 and -3069 of the cathepsin D promoter were used as control (lower panel).The 1 kb DNA ladder is shown to the left. (D) SiRNA knock down of endogenous SPBP in R1881 stimulated LNCaP cells reduces the expression of the AR target gene PSA. LNCaP cells were transfected with SPBP siRNA or scrambled siRNA, stimulated with R1881 for 48 hours before harvesting. RT-PCR reactions were run on PSA and β-actin mRNA. The average amount of PSA mRNA correlated to β-actin mRNA based on four independent experiments are shown with standard deviations. The right panel shows the knock-down of SPBP expression in LNCaP cells transfected with SPBP siRNA compared to scramled siRNA transfected and untransfected LNCaP cells.

Subconfluent HEK293 or LNCaP cells in 24-well tissue culture dishes (Becton Dickinson) were transiently transfected using Metafectene Pro (Biontex) according to the manufacturer's protocol. The cells were cultured in 5% charcoal treated serum one day before transfection. Medium with 0.5% charcoal treated serum and 10^−7^ M synthetic androgen R1881 (Perkin-Elmer) was (if indicated) added to the cells 1 hour before transfection. All wells were cotransfected with 75 ng pDestHA-AR, 75 ng of the luciferase reporter p285PB-Luc [31], and 5 ng pCMV-β-galactosidase (Stratagene). In addition, the HEK293cells were cotransfected with 0–25 ng pDestHA-Pax6, and 50 or 150 ng pDestHA-SPBP, as indicated in Figure 1A. The LNCaP cells were in addition cotransfected with 5–200 ng pDestHA-Pax6 as indicated in Figure 1B. pcDNA3HA (Invitrogen) was used to equalize the concentration of DNA in each transfection. Extracts were prepared 20 hours after transfection using a Dual-Light luciferase and β-galactosidase reporter gene assay system (Tropix), and subsequently analyzed in a Labsystems Luminoskan RT dual injection luminometer. The assays were performed in triplicates and repeated at least three times.

### Immunoblotting of whole cell extracts

Whole cell extracts were prepared by adding 400 µl lysis buffer (1% SDS; 10 mM TrisHCl pH 7.5; 90°C) to confluent cells in 10 cm culture dishes (Nunc). The lysates were sonicated, centrifuged, and stored at −70°C. The protein concentration was measured with a BioRad DC protein assay kit. Fifty µg of each lysate was run on 6 or 12% SDS-polyacrylamide gels (SDS-PAGE) in Tris-Glycine buffer (25 mM Tris, 250 mM glycine, 0.1% SDS). Biotinylated Protein Ladder (Cell Signalling) was used as a molecular weight marker. The proteins were subsequently immunoblotted on Hybond-ECL nitrocellulose membranes (Amersham) in Towbin buffer (25 mM Tris, 192 mM glycine, 20% methanol) at 100 V for 1 hour. The membranes were blocked in 5% non-fat dried milk for 1 hour at room temperature. Primary antibody was added overnight at 4°C. The primary antibodies used were rabbit anti-Pax6 (1∶1.000, Millipore), mouse anti-AR (1∶100, Santa Cruz), rabbit anti-SPBP (1∶500, [10]), mouse anti-HA (1∶1.000, clone 12CA5, Roche) and rabbit anti-β-actin (1∶1.000, Sigma-Aldrich). Secondary antibodies, HRP-conjugated anti-mouse or anti-rabbit IgG (1∶2.000, Pierce) and anti-Biotin (1∶2.000, Cell Signalling), were added for 1 hour at room temperature. Detection was performed using Western blot luminol reagent (Santa Cruz Biotechnology) and a LumiAnalyst imager (Roche Applied Sciences).

### Chromatin Immunoprecipitation

Subconfluent HeLa cells in 10 cm dishes were transiently transfected with 2.25 µg of p285-PBLUC [31], pDestHA-AR and pDestHA-SPBP using Metafectene Pro (Biontex). In addition, some dishes were co-transfected with 300 ng or 1.5 µg pDestHA-Pax6 as indicated in Figure 1D. Five hours post transfection the cells were stimulated by 10^−7^ M synthetic androgen R1881 (Perkin-Elmer). Sixteen hours post transfection the cells were harvested and chromatin immunoprecipitation was performed essentially as described previously [10]. Primers used to amplify precipitated probasin promoter were 285PB-Luc-fw and 285PB-Luc-rev (Table S2).

### Real-Time PCR

Subconfluent LNCaP cells in 6 well dishes were tansfected with 20 nM SPBP siRNA [10] or scrambled siRNA [10] using RNAiMAX (Invitrogen). The cells were stimulated by R1881 (10^−7^ M) for 48 hours post transfection. RNA was isolated using RNAeasy Plus Minikit (QIAGEN), cDNA made using Transcriptor Universal cDNA Master (Roche), and RT-PCR performed on a STRATAGENE ×300 amplification system using FastStart Universal SYBR Green Master (Roche). Primers were PSA-fw, PSA-rev, Actin-fw and Actin-rev (Table S2). The reactions were run for an initial step at 95°C for 10 min, followed by 40 cycles of amplification at 95°C for 15 s and 60°C for 1 min. All data were collected during the extension step, and a melting curve was obtained at the end of the PCR reaction to verify that only one product was produced.

### Confocal microscopy

GFP-AR expression was induced from the GFP-AR 3108 cell line by removing the tetracycline from the medium 3 days before the experiments were performed. G418 and puromycin were also removed from the medium to improve the expression. The cells were cultured in 8-chambered cover slides (Nunc) and transiently transfected with 100 ng pDestCherry-Pax6 or 150 ng pDestCherry-SPBP using TransIT-LT1 (Mirus Bio) according to the manufacturer's protocol. As described previously, the cells were starved in medium with charcoal-treated serum and hormone induced with R1881. Subconfluent HeLa cells in 8-well coverglass slides were transiently co-transfected with 175 ng pDestEGFP-SPBP and 25 ng pDestmCherry-Pax6 using TransIT-LT1. Live cell images were taken the following day after transfection using a Zeiss LSM510 confocal laser scanning microscope. Colocalization was studied using the Zeiss LSM 510 software.

### Coimmunoprecipitation

For coimmunoprecipitation of AR and Pax6, expression of 3×Flag-Pax6 was induced from HeLa FlpIn 3×Flag-Pax6 cells by adding 1 µg/ml tetracycline. Subconfluent cells in 10 cm dishes (Nunc) were transiently transfected with either pDestEGFP-AR or pEGFP-C1 using Metafectene Pro (Biontex) according to the manufacturer's protocol. R1881 (10^−7^ M) was added to stimulate the AR. For coimmunoprecipitation of AR and SPBP, 2×10^5^ HeLa cells were seeded in 10 cm dishes in MEM supplied with 5% charcoal treated serum, and incubated for 18–20 hours. The cells were then transiently cotransfected with 3 µg pDestEGFP-AR and 7 µg pDestHA-SPBP, using the Calcium Phosphate transfection procedure [32]. 3–4 hours after transfection, the cells were stimulated using R1881 (10^−7^ M). The cells from both experiments were harvested 20 hours post transfection and immonoprecipitaed essentially as described [10]. Primary antibodies used in the western blot were mouse anti-Flag M2 (1∶5.000; Stratagene), rabbit anti-SPBP (1∶500, [10]) or rabbit anti-GFP (1∶1.000; Abcam).

For *in vitro* coimmunoprecipitation assays, expression vectors pDestHA-SPBP and pDest53-AR were *in vitro* translated in the presence of ^35^S-methionine and immunoprecipiated as described [10].

### Fluorescence resonance energy transfer (FRET)

FRET with photobleaching of the acceptor was done using a Zeiss LSM510 META confocal microscope as described [20]. HeLa cells were cotransfected with 50 ng of both CFP- and YFP-tagged expression constructs using LipofectAmine PLUS (Invitrogen). Three to five cell nuclei expressing a 1∶1 ratio of the CFP-and YFP-tagged proteins were examined per protein pair. A CFP-YFP fusion protein with a linker of 52 residues (pDestECFP-re7-EYFP), and a CFP-Pax6-YFP fusion protein (pDestECFP-Pax6-EYFP) were used as positive FRET controls. The following protein pairs were used as negative controls: CFP (pDestECFP-C1) and YFP (pDestEYFP-C1), CFP-AR (pDestECFP-AR) and YFP, and CFP and Pax6-YFP (pDestPax6-EYFP). Percent FRET efficiency between CFP- and YFP-tagged proteins was calculated as the difference between CFP fluorescence before and after bleach using the formula: ((A1–A0)/A1)–((B1–B0)/B1)/BkYFP-Pax6×100%. A1 and A0 are CFP emission in the region of interest (ROI) in the bleached cell after and before photobleaching, while B1 and B0 are CFP emission in a non-bleached ROI in a control cell after and before photobleaching, respectively [33]. The bleach constant Bk for YFP-Pax6 was experimentally found to be 0.68.

### GST and MBP pulldown assays

GST and GST fusion proteins were expressed in *Escherichia coli* BL21STAR (λDE3) pLysS (Novagen) and purified from extracts using glutathione-sepharose 4 fast flow beads (Amersham Biosciences). MBP and MBP fusion proteins were expressed in *E. coli* DH5α and purified on amylose resin (New England Biolabs). *In vitro* translation and dilution of ^35^S-labeled proteins were performed as described for *in vitro* coimmunoprecipitation. GST or MBP bound to beads were added to the samples for 30 minutes at 4°C on a rotating wheel to remove unspecific binding. The supernatants were subsequently mixed with 1–2 µg of the indicated fusion proteins immobilized on beads and incubated at 4°C for 1 hour. After 5 times washing in NET-N buffer, the beads were resuspended in 2× SDS gel loading buffer and boiled for 5 minutes. If indicated, 10^−7^ M synthetic androgen R1881 was added to the reactions. The samples were separated on 10% SDS-PAGE gels together with a molecular weight marker (New England Biolabs #P7703S), stained with Coomassie brilliant blue (CBB) and vacuum dried. Signal from ^35^S-labeled proteins was detected with a Fujifilm bioimaging analyzer FUJI-BAS5000.

## Results

### Pax6 represses SPBP-mediated enhancement of AR activity

Recent reports show Pax6 to be a repressor of AR activity [29], and the *PAX6* gene to be hypermethylated in prostate cancer cells [34]. p285PB-Luc contains a fragment of the AR targeted rat probasin promoter upstream of the luciferase gene [31]. Using p285PB-Luc as a reporter, we confirm that Pax6 represses AR-mediated transactivation in LNCaP prostate cancer cells (Figure 1A). These results correlate well with other reports suggesting Pax6 to have a tumor suppressor function [24]–[28].

We have previously found that the nuclear protein SPBP acts as a coactivator of Pax6 [8]. Interestingly, SPBP also interacts with phosphorylated ERα and represses its transcriptional activity [11]. These results encouraged us to investigate whether Pax6 and SPBP together would have any impact on AR activity. To this end, luciferase reporter gene assays were performed in HEK293 cells using p285PB-Luc as a model system. Figure 1B shows that SPBP alone enhanced the transcriptional activity of AR in a dose dependent manner, hence acting as a transcriptional coactivator of AR. Coexpression of SPBP and increasing concentrations of Pax6 resulted in corresponding increased repression of the SPBP-mediated enhancement of AR activity. However, increasing ectopic expression of SPBP seems to partly restore the activity suppressed by Pax6, suggesting a competitional recruitment to AR.

Chromatin immunoprecipitation assays were performed to investigate whether SPBP was recruited to the AR target promoter probasin, and if Pax6 could affect this recruitment. In order to obtain expression of the three proteins in the same cells, with varying amounts of Pax6, HeLa cells were cotransfected with the probasin-luciferase promoter construct and vectors expressing AR, SPBP and/or Pax6. The results presented in Figure 1C (upper panel) clearly show that SPBP and AR are associated with the same fragment of the probasin promoter. Furthermore, expression of increasing amounts of Pax6 reduces the amount of SPBP associated with the promoter. An upstream fragment of the Cathepsin D promoter was amplified on the same samples to verify specificity (Figure 1C, lower panel). Hence, Pax6 seems to inhibit recruitment of SPBP to the AR target probasin promoter.

As shown in Figure 1B, SPBP enhances AR activity only modestly in reporter gene assays using exogenous proteins. To determine whether SPBP has an effect on the expression of an endogenous AR target gene, SPBP siRNAs were transfected into LNCaP cells followed by monitoring the expression level of PSA mRNA by RT-PCR. SPBP expression is highly knocked down by the SPBP siRNAs (Figure 1D, right panel). Importantly, PSA mRNA is reduced to approximately 40% in the SPBP knock down cells compared to scrambled siRNA (Figure 1D, left panel). These results show that SPBP enhances expression of AR target genes also in an endogenous system. The modest effect of SPBP in the reporter gene system may be due to low expression levels of exogenous SPBP compared to exogenous Pax6 and AR (Figure S1).

Together, these results show that SPBP acts as a potent transcriptional coactivator of AR, while Pax6 acts as a repressor inhibiting recruitment of SPBP to the AR target promoter and thereby reduces the SPBP-mediated enhancement of AR activity.

### AR, SPBP and Pax6 display a very similar nuclear distribution pattern in androgen stimulated cells

**Figure 2:**
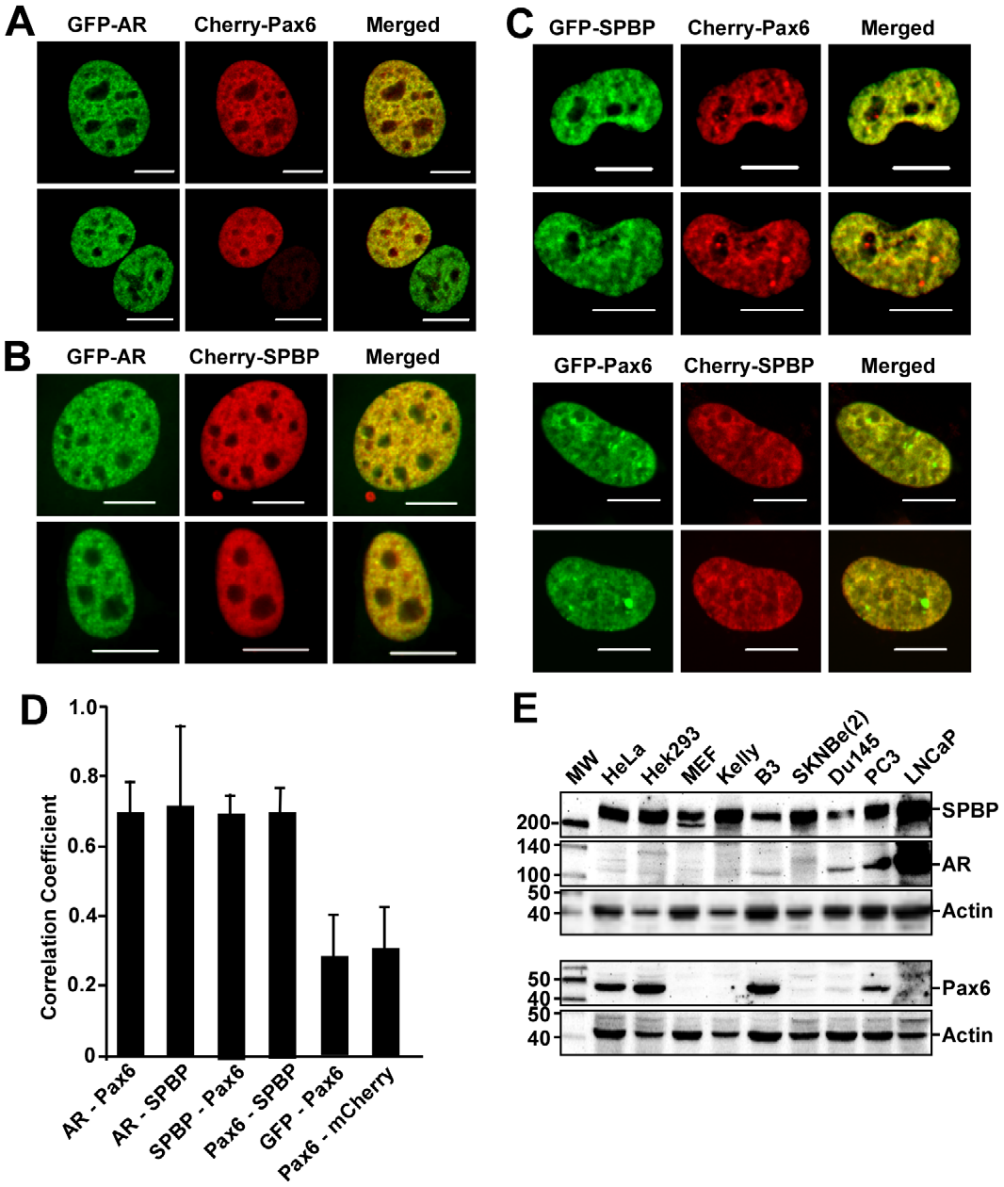
Colocalization and coexpression of AR, Pax6 and SPBP. (A) and (B) Colocalization between GFP-AR and Cherry-Pax6 or Cherry-SPBP, respectively. The GFP-AR 3108 expressing cell line [30] was transiently transfected with 100 ng pDestCherry-Pax6 or 150 ng pDestCherry-SPBP using TransIT-LT1 (Mirus Bio). The cells were stimulated with synthetic androgen R1881 and live cell images obtained using a Zeiss LSM510 confocal laser scanning microscope. Scale bars: 10 µm. (C) Colocalization between GFP- and Cherry-tagged SPBP and Pax6. HeLa cells were transiently cotransfected with either 175 ng pDestEGFP-SPBP and 25 ng pDestCherry-Pax6, or 25 ng pDestEGFP-Pax6 and 175 ng pDestCherry-SPBP using TransIT-LT1 (Mirus Bio). Live cell images were obtained as in A. Scale bars: 10 µm. (D) Pearson's correlation coefficients support the colocalization of GFP-AR with mCherry-SPBP and mCherry-Pax6 shown in A and B. The correlation is based on the average of 5–10 independent cells, with standard deviations shown. The nuclear correlation between mCherry-Pax6 and nuclear GFP, and GFP-Pax6 and nuclear mCherry, were used as negative controls. (E) Coexpression of Pax6, SPBP and AR in mammalian cell lines. Approximately 50 µg proteins from each of the whole cell extracts were separated on 6 and 12% SDS-polyacrylamide gels, followed by immunoblotting using anti-Pax6 (Chemicon), anti-AR (Santa Cruz), anti-SPBP [10] and anti-actin (Sigma) antibodies. Actin was used as a loading control. The cell lines used were HeLa (human epithelial), HEK293 (human embryonic kidney), MEF (mouse embryonic fibroblast), Kelly and SKNBe(2) (human neuroblastoma), and Du145, PC3 and LNCaP (human prostate cancer).

To further evaluate the putative association among Pax6, SPBP and AR, their nuclear distribution patterns were investigated by confocal laser scanning microscopy. The mammary adenocarcinoma cell line GFP-AR 3108 stably expressing GFP-AR [30] was transiently transfected with plasmids expressing Cherry-Pax6 or Cherry-SPBP (Figure 2A and 2B, respectively). Both Cherry-Pax6 and Cherry-SPBP strongly colocalized with GFP-AR in the nucleus of nearly all 3108 cells upon synthetic androgen (R1881) stimulation. The proteins are located throughout the nucleus except from the nucleolus, in a subnuclear localization pattern we have previously denoted as chromatin-rich, nuclear territories [35]. Similar distribution patterns were observed in HeLa cells overexpressing EGFP-AR and Cherry-Pax6 or Cherry-SPBP (data not shown). In line with this, Pax6 and SPBP were found to colocalize almost completely in the nucleus of all transfected HeLa cells (Figure 2C). Lack of available Pax6 and SPBP antibodies that work well in immunostaining, made it difficult to study the distribution pattern of the endogenous proteins. However, colocalization was assayed on cells that displayed moderate levels of the exogenous proteins. Furthermore, calculation of the Pearson's correlation coefficient of several cells resulted in an average correlation coefficient of 0.7 for Pax6 and AR, SPBP and AR, and Pax6 and SPBP (Figure 2D). Taken together, these results strongly suggest that AR, Pax6 and SPBP are enriched in the same chromatin-rich territories of R1881 stimulated human cells.

To investigate whether Pax6, SPBP and AR are coexpressed in cells, whole cell extracts from nine different cell lines were analyzed by immunoblotting using antibodies against Pax6, SPBP and AR. The blots in Figure 2E show that SPBP is expressed in all of the cell lines tested, while Pax6 is expressed in HeLa, HEK293, B3 and PC3 cells. Expression of Pax6 in the B3 cell line was not unexpected, given that it is a human lens epithelial cell line. We also detected Pax6 at very low levels in Du145 and the human neuroblastoma cell line SKNBe(2), while no expression was detected in the mouse embryonic fibroblasts (MEFs) or the human prostate cancer cell line LNCaP. In contrast, high expression of AR was, as expected, detected in the latter cell line. Human Du145 and PC3 prostate cancer cells are frequently used as AR negative control cells, but we detected some expression of AR in these cell lines as well. AR was also expressed in HeLa, HEK293, Kelly and B3 cells at very low levels, but was not detected in the MEF or SKNBe(2) cell lines. In summary, Pax6, SPBP and AR are coexpressed in the B3 lens epithelial and PC3 prostate cancer cell lines. Interestingly, SPBP is highly expressed in the AR positive prostate cancer cell line LNCaP. SPBP is also expressed in glandular cells of the prostate and in 30% of prostate cancer samples in the Human Protein Atlas (www.proteinatlas.org). This correlates well with the expression pattern of AR and several AR target genes such as PSA, Elk4 (Sap-1a) and the AR-coactivator LSD1 (www.proteinatlas.org, [36]).

### FRET analysis reveals a direct interaction between AR and Pax6 in cells

**Figure 3:**
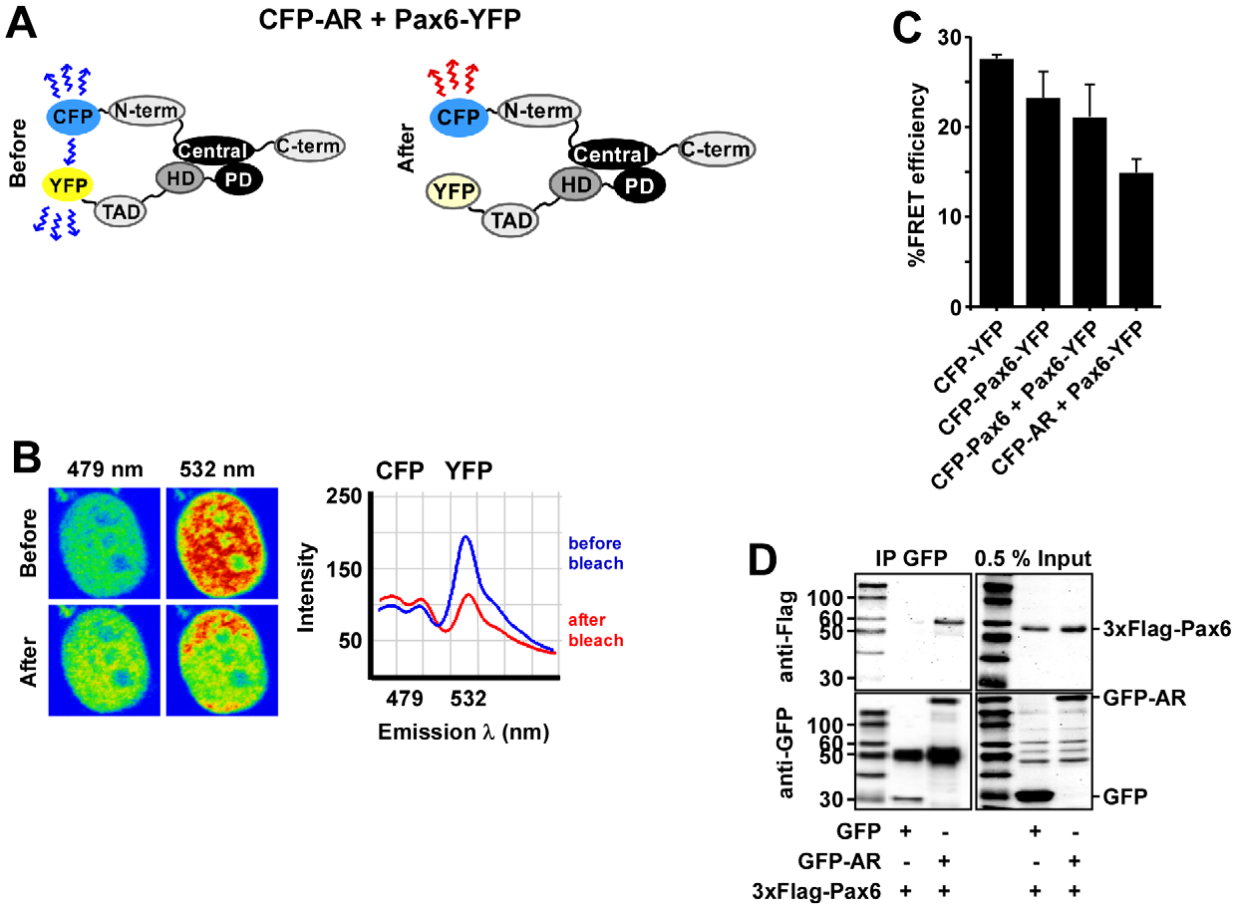
Pax6 and AR interact directly *in vivo*. (A–C) FRET between Pax6 and AR. HeLa cells transiently expressing a 1∶1 ratio of CFP-AR (pDestECFP-AR) and YFP-Pax6 (pDestPax6-EYFP) were subjected to FRET as described in the methods section. A schematic illustration of CFP-AR and YFP-Pax6 before and after acceptor photobleaching is presented in A. The cell images in B are visualized in pseudo-colors before and after acceptor photobleaching. The YFP acceptor was bleached, and FRET detected as decreased YFP-emission at 532 nm and a corresponding increased CFP-emission at 479 nm in the bleached area. The graphical display in B shows the emission spectrum of CFP-AR together with YFP-Pax6 at 479 and 532 nm, respectively, before (blue) and after (red) acceptor photobleaching. (C) Percent FRET between CFP-AR and Pax6-YFP compared with FRET between CFP-YFP, CFP-Pax6-YFP, and CFP-Pax6 - Pax6-YFP. The FRET efficiency was calculated as described in the methods section. Each bar represents the mean of 3–5 experiments. (D) Coimmunoprecipitation of GFP-AR and 3×Flag-Pax6. HeLa FlpIn 3×Flag-Pax6 cells were induced to express 3×Flag-Pax6 and subsequently transfected with pDestEGFP-AR or pEGFP-C1 using Metafectene Pro (Biontex). A GFP-antibody (Abcam) was used to immunoprecipitate GFP-AR-3×Flag-Pax6 complexes from the cells. The upper right gel shows the 0.5% input of 3×Flag-Pax6 and the lower right gel the 0.5% input of GFP and GFP-AR. The upper left gel shows that 3×Flag-Pax6 was immunoprecipitated together with GFP-AR, but not with the GFP control.

Recently, an association between Pax6 and AR in COS-1 cells has been reported [29]. To evaluate if this interaction is direct in human cells, FRET experiments were performed using the acceptor photobleaching method as previously described [20], [33]. HeLa cells were cotransfected with expression constructs for Pax6 and AR tagged with yellow (YFP) and cyan (CFP) fluorescent proteins, respectively. With the CFP and YFP fluorescent proteins, FRET only occurs within a distance of 10 nm [37]. The CFP-AR and Pax6-YFP fusion proteins are schematically illustrated in Figure 3A before and after photobleaching. As shown in Figure 3B, strong FRET was detected between Pax6 and AR in the nuclei of HeLa cells. Figure 3C shows the calculated percent FRET efficiencies. A FRET efficiency of 21±3.7% was detected between the CFP-Pax6 and Pax6-YFP fusion proteins, while the FRET efficiency between Pax6-YFP and CFP-AR was 14.8±1.5%. A CFP-YFP fusion protein with a linker of 52 non-specific amino acids separating CFP and YFP was used as a positive control, yielding a FRET efficiency of 27.5±0.4%. Another positive control, CFP-Pax6-YFP, gave a FRET efficiency of 23.3±2.9%. CFP versus YFP, CFP-AR versus YFP, and CFP versus Pax6-YFP were used as negative controls and displayed little or no FRET (data not shown). The results from the FRET experiments clearly show that Pax6 and AR bind directly to each other in the nucleus of living human cells. This was further confirmed by coimmunoprecipitation of EGFP-AR and 3×Flag-Pax6 from HeLa FlpIn cells (Figure 3D). Expression of Flag-tagged Pax6 was induced by adding tetracycline to the medium, before the cells were transiently transfected with plasmids expressing EGFP-AR. AR was stimulated by adding synthetic androgen to the medium, and a GFP-antibody was used to immunoprecipitate GFP-AR-3×Flag-Pax6 complexes from the cells. The upper left panel in Figure 3D shows that 3×Flag-Pax6 was immunoprecipitated with GFP-AR, but not with the GFP alone. This further confirms the AR-Pax6 interaction established with the FRET assays.

### The PD of Pax6 binds to the DBD of AR

**Figure 4:**
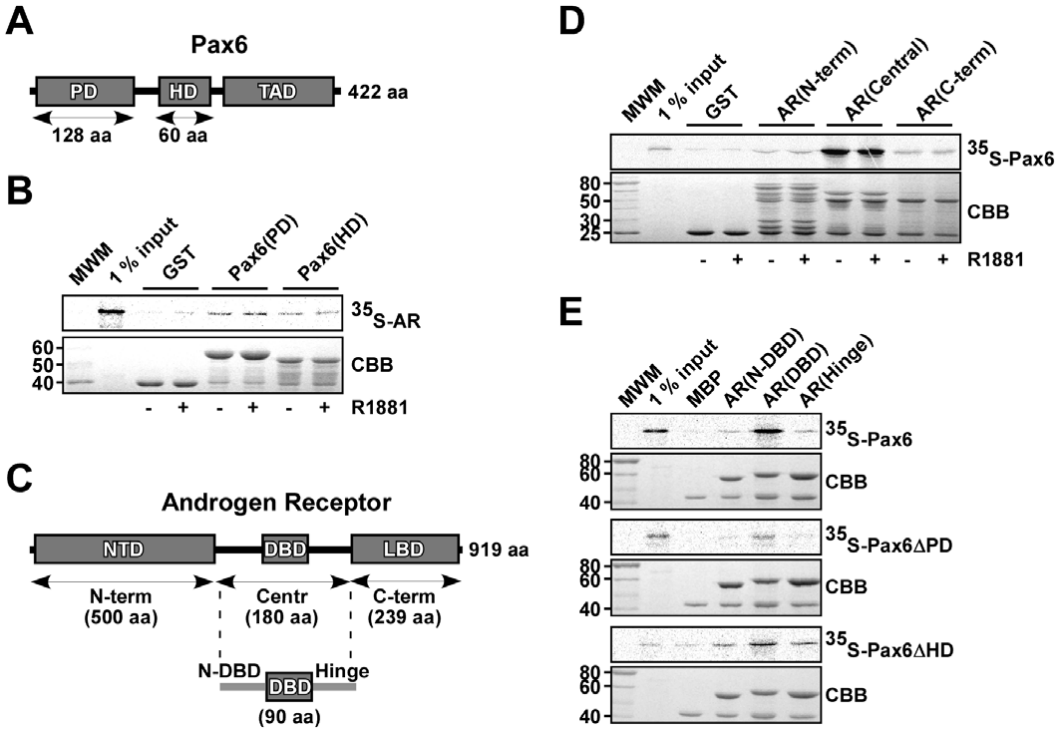
Mapping of the Pax6-AR interaction *in vitro*. (A) Schematic illustration of Pax6. Pax6 consists of two DNA-binding domains (DBDs), the N-terminal paired domain (PD), the paired-type homeodomain (HD) and a C-terminal transactivation domain (TAD). (B) AR interacts with the DBDs of Pax6. GST, GST-Pax6(PD) and GST-Pax6(HD) were immobilized on glutathione-sepharose beads and used to pull down *in vitro* translated ^35^S-methionine labeled AR in the presence or absence of 10^−7^ M R1881. The samples, 1% input, and a molecular weight marker (MWM) were run on 10% SDS-polyacrylamide gels and stained with Coomassie brilliant blue (CBB) (lower panel). Signal from ^35^S-labeled proteins was detected with a Fujifilm bioimaging analyzer FUJI-BAS5000 (upper panel). (C) Schematic illustration of AR. The N-terminal domain (NTD), the central DBD, and the C-terminal ligand-binding domain (LBD) are illustrated. The arrows indicate the different deletion constructs made, with their size in number of amino acids given in parenthesis. (D) Pax6 binds to the central part of AR. GST, GST-AR(N-term), GST-AR(Central) and GST-AR(C-term) immobilized on glutathione sepharose beads were used to pull down *in vitro* translated ^35^S-methionine labeled Pax6 in the presence or absence of 10^−7^ M R1881. Upper panel shows the amount of ^35^S-labeled Pax6 pulled down, and the lower panel shows the CBB staining of the GST fusion proteins. (E) Further mapping of the Pax6-AR interaction shows that Pax6 interacts with the DBD of AR. The interaction is reduced by deleting the PD of Pax6, but not by deleting the HD. The indicated AR constructs fused to MBP were used to pull down *in vitro* translated ^35^S-methionine labeled Pax6, Pax6ΔPD and Pax6ΔHD as described. The upper gel pictures show the detected signal from ^35^S-labeled proteins, and the lower gel pictures show the CBB staining of the MBP fusion proteins. All results are representative of three independent experiments.

Next, pulldown assays were performed in order to map the interacting regions of AR and Pax6. The various domains of both proteins, as indicated in Figure 4A and C, were expressed as GST or MBP fusion proteins in *E. coli*, while the full-length proteins were *in vitro* translated in the presence of ^35^S-methionine. The GST pulldown assay in Figure 4B shows that AR binds to both the isolated PD and the HD of Pax6, but with highest affinity to the PD. Adding synthetic androgen (R1881) did not affect the interactions significantly. Neither did addition of benzonase (Figure S2). Hence, the interactions are not dependent on DNA-binding.

To map the region of AR responsible for mediating the reaction, three various AR deletions were constructed; the 500 amino acids long NTD (N-term), the 180 amino acids long central part including the DBD and the hinge region (Central), and the 239 amino acids long C-terminal LBD (C-term) (Figure 4C). The GST pulldown assay in Figure 4D shows that full-length Pax6 interacts strongly with the central part of AR. Again, adding synthetic androgen did not affect the binding. The interaction between AR(central) and Pax6 is further strengthened by pulldown assays showing that also other Pax proteins interact with this part of AR (Figure S3).

The central region of AR was further split in the 50 amino acids N-terminal to the DBD (N-DBD), the 90 amino acids long DBD, and the 52 amino acids hinge region (Figure 4C). MBP fusion proteins of the three constructs were used to pull down *in vitro* translated Pax6. Compared to the flanking regions, full-length Pax6 interacted strongly with the DBD of AR (Figure 4E, upper panels). Deleting the PD of Pax6 (Pax6ΔPD) reduced this interaction significantly (Figure 4E, middle panels), while deleting the HD (Pax6ΔHD) did not affect the interaction (Figure 4E, lower panels). In conclusion, the interaction between AR and Pax6 is mainly between the DBD of AR and the PD of Pax6, and is neither dependent on androgens nor DNA.

### SPBP interacts with the DBD of AR

**Figure 5:**
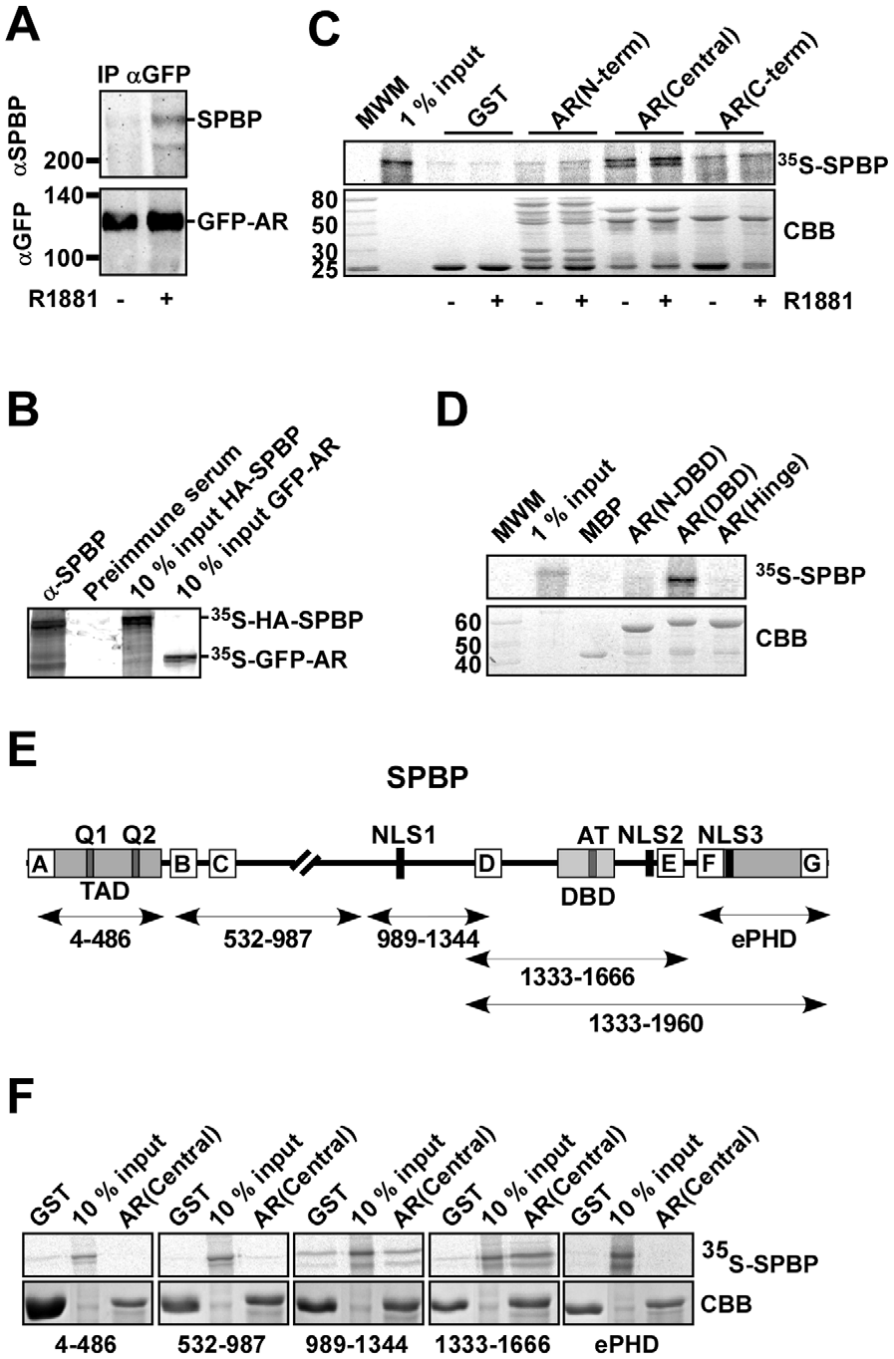
AR and SPBP interact in cells and *in vitro*. (A) SPBP coprecipitates with AR in R1881-stimulated HeLa cells. pDestEGFP-AR and pDestHA-SPBP were cotransfected into HeLa cells grown in medium with or without R1881. The proteins were precipitated using an anti-GFP antibody (Abcam), separated by SDS-PAGE and visualized by anti-SPBP antibody [10] (upper panel) and anti-GFP antibody (lower panel). (B) SPBP and AR interact *in vitro*. HA-SPBP and GFP-AR were *in vitro* translated in the presence of ^35^S-methionine and immunoprecipitated using anti-SPBP antibody or preimmune serum. Precipitated complexes and 10% input of the *in vitro* translated proteins were resolved by SDS-PAGE. (C) SPBP interacts with the central part of AR. The indicated AR deletion constructs were expressed as GST fusion proteins, and used to pull down *in vitro* translated ^35^S-methionine labeled SPBP. Upper panel shows the ^35^S-labeled proteins, and lower panel CBB staining of the GST fusion proteins. (D) SPBP interacts with the AR(DBD). The central part of AR was divided into three; DBD, N-DBD and hinge. MBP fusions of the indicated AR constructs were used to pull down ^35^S-methionine labeled SPBP. Upper panel shows ^35^S-labeled SPBP, while lower panel shows CBB staining of the MBP fusion proteins. (E) Schematic illustration of SPBP. SPBP contains an N-terminal TAD, a DBD containing an AT-hook motif (AT), a C-terminal extended plant homeodomain (ePHD), three nuclear localization signals (NLS) and two glutamine rich regions (Q1 and Q2). The boxes labeled A-G represent conserved regions. SPBP constructs used in this study are indicated with arrows and number of amino acids. (F) Amino acids 1333–1666 of SPBP interact with the central part of AR *in vitro*. The indicated regions of SPBP were *in vitro* translated and used in pull down assays with GST or GST-AR(Central). Coprecipitations were detected as described in D. All results are representative of three independent experiments.

Since SPBP is reported to interact with ERα and here is found to function as a coactivator of AR, we asked whether SPBP was able to associate with AR in cells. HeLa cells were transiently cotransfected with EGFP-AR and HA-SPBP expressing plasmids. An anti-GFP antibody was used to precipitate EGFP-tagged AR from cell lysates, and immunoblotting performed using an anti-SPBP antibody [10]. The upper panel in Figure 5A shows that SPBP coprecipitates with EGFP-AR in HeLa cells stimulated with R1881, but not in unstimulated HeLa cells. The lower panel in Figure 5A confirms precipitation of EGFP-AR in extracts both from stimulated and unstimulated HeLa cells. This strongly suggests that SPBP interacts with AR in the nucleus of human cells.

The interaction between the full length proteins were confirmed by coimmunoprecipitation of *in vitro* translated proteins (Figure 5B), followed by mapping of the interacting regions using pulldown assays. First, various regions of AR (schematically illustrated in Figure 4C) were expressed and purified from *E. coli* as GST or MBP fusion proteins while full-length SPBP were produced *in vitro* in the presence of ^35^S-methionine. The pulldown assays in Figure 5B and 5C show that SPBP, like Pax6, interacts with the central region, and more specifically the DBD, of AR. A weak interaction between SPBP and the C-terminal part of AR was also detected. The interactions are not affected by the presence or absence of synthetic androgen.

SPBP was then split in parts, as indicated in Figure 5D, *in vitro* translated and used in pulldown assays together with the central part of AR fused to GST. The results of these pulldowns show that amino acids 1333–1666 of SPBP exclusively interacted with AR(Central) (Figure 5E). Interestingly, the 1333–1666 region of SPBP contains its DBD and also the region (1576–1601) that mediates the interaction with ERα [11]. In addition, this region encompasses three highly conserved motifs (D-box, E-box, and AT-hook). To determine whether DNA binding was important for the SPBP-AR interaction to take place, benzonase was included in some of the GST pulldown assays. However, adding this endonuclease did not have any impact on the binding (Figure S2). Hence, here we have mapped a DNA-independent interaction between AR and SPBP, mediated by the DBD of AR and the 1333–1666 region of SPBP containing its DBD.

### Pax6 and SPBP compete for binding to AR(DBD)

**Figure 6:**
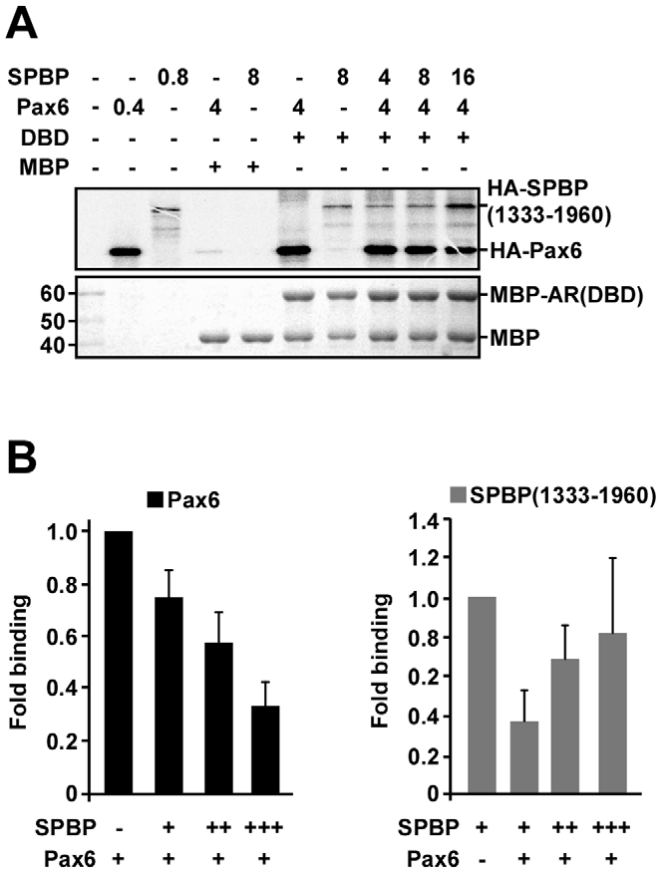
Pax6 and SPBP compete for binding to AR(DBD). (A) MBP-AR(DBD) immobilized on amylose resin beads were incubated with constant amounts of ^35^S-labeled HA-Pax6 and increasing amounts of HA-SPBP(1333–1960). The upper gel picture shows the signal detected for the ^35^S-labeled proteins, and the lower gel picture shows the amount of MBP fusion proteins (CBB staining). The numbers above the upper gel picture indicate how many µl that were used of each *in vitro* translated protein. The presented result is representative of three independent experiments. (B) Quantification based on the average fold binding of three independent experiments. The quantification was performed using Image Gauge version 4 from FUJI. Left panel: The amount of Pax6 bound to MBP-AR(DBD) was set to 1 fold binding. Increasing the concentration of SPBP while keeping the amount of Pax6 constant, results in reduced binding of Pax6 to the AR(DBD). Right panel: The amount of SPBP bound to MBP-AR(DBD) was set to 1 fold binding. Coincubation of Pax6 and SPBP with AR(DBD) reduces the amount of SPBP bound to AR. The binding is rescued by increasing amounts of SPBP, while keeping the input of Pax6 constant.

The findings that coexpression of Pax6 repressed SPBP-mediated transactivation of AR activity, inhibited association of SPBP with an AR target promoter, and that both SPBP and Pax6 interacted with the same domain of AR, prompted us to investigate whether there could be a competition between Pax6 and SPBP for AR(DBD) binding. In an attempt to study this, MBP pulldown assays were performed with increasing amounts of SPBP(1333–1960) together with constant amounts of full length Pax6 and MBP-AR(DBD) immobilized on amylose resin. The 1333–1960 region of SPBP binds equally well to AR(DBD) as the full length protein (data not shown), and was therefore used in this assay to reduce the migration differences between Pax6 and SPBP. As shown in Figure 6, Pax6 and SPBP may in fact compete for binding to AR(DBD). Increasing amounts of SPBP reduce the amount of Pax6 coprecipitated with MBP-AR(DBD) while the amount of SPBP binding to AR(DBD) is increased. On the other hand, presence of Pax6 reduces the amount of SPBP bound to AR(DBD) (Figure 6B, right). Taken together, these results suggest that there is a competition between Pax6 and SPBP for binding to AR, and that this competition is a possible mechanism for the repressive effect Pax6 has on SPBP-mediated transactivation of AR activity.

## Discussion

AR is critically dependent on recruitment of coactivators and corepressors for transcriptional regulation of its target genes. The differential interaction of AR with various cofactors appears to control the fine balance between proliferation and differentiation important for maintenance of the normal prostate. Changes in cofactor levels may shift the balance between suppressing or facilitating cancer progression (reviewed in [38]–[40]). In this study, we have identified the transcriptional coregulator SPBP as a coactivator of AR, stimulating AR-mediated transcription of the probasin promoter and enhancing the expression of the AR target gene PSA in androgen stimulated LNCaP cells. SPBP is expressed in most cell lines, but highly expressed in the androgen dependent cancer cell line LNCaP. In normal prostate tissue, both SPBP and AR are found in glandular cells (www.proteinatlas.org), and we show that overexpressed AR and SPBP have very similar nuclear distribution patterns in cells stimulated with androgen. Interestingly, 30% of the prostate cancer samples in the Human Protein Atlas (www.proteinatlas.org) display a significant enrichment of nuclear SPBP. Since coregulators of nuclear hormone receptors often have the ability to influence the activity of multiple receptors [38], the AR-SPBP association is strengthened by the previously reported interaction between SPBP and phosphorylated ERα [11].

The developmental transcription factor Pax6 was recently reported to act as a repressor of androgen stimulated AR activity [29]. Here we show that Pax6 in addition inhibits SPBP-mediated stimulation of AR activity and the association of SPBP with an AR target promoter. Both Pax6 and SPBP were found to associate with AR in androgen stimulated cells, and their distribution patterns in the cell nucleus were completely overlapping. Mapping of interacting regions revealed that all three proteins use their DBDs to mediate the interactions. Pax6 contains two DBDs, the PD and the HD. Although both domains are able to interact with AR, the PD seems to bind more strongly. The interaction between Pax6 and AR(DBD) correlates well with the fact that other Pax proteins also interact with the central region of AR. It also correlates with a recent report showing that the HD protein HoxB13 represses AR activity by interacting with the AR(DBD) [41]. The interacting region was in this case mapped using AR(DBD) point mutations in a mammalian two-hybrid assay.

Interestingly, our *in vitro* competition assays indicated that SPBP and Pax6 compete for AR(DBD) binding. Several AR coactivators are reported to interact with the AR(DBD) (reviewed in [38], [39]). Our results suggest that the inhibitory effect of Pax6 on AR-mediated transcriptional activity is obtained by masking coactivator binding sites in or displacing coactivators binding to the DBD of AR. This is in line with recent reports on the homeoproteins HOXB13 [41] and HOXC8 [42]. Both proteins act as repressors of AR-mediated transcriptional activity. HOXB13 significantly reduced the recruitment of AR coactivators to specific AR target genes, while HOXC8 was shown to block AR-dependent recruitment of the coactivators SRC-3 and CBP. They also found that overexpression of SRC-3 reverses the HOXC8-mediated blockage. This is similar to our results, demonstrating that increased SPBP expression partly restores the Pax6-mediated inhibition.

Understanding the detailed mechanisms and mapping the specific amino acids important for these interactions and competitions may have a potential in the future development of targeted therapies against prostate cancer. AR plays an important role in both early and advanced stages of prostate cancer etiology. Formation of active AR-directed transcription complexes occurs via a sequential recruitment of coactivators [38]–[40] and alteration in expression and function of an expanding number of cofactors are suggested to underlie pathological conditions. Hence, targeting coregulators for new therapeutics represent a new opportunity (reviewed in [38]). Importantly, cofactors have been suggested to play important roles in the development of androgen insensitive prostate cancer [43], by enhancing the ability of AR to maintain sufficient function in a low androgen environment for maintenance of cell growth and survival. A putative strategy to inhibit or diminish recruitment of coactivators to the receptor is to mimic the AR-Pax6 interaction in cancer cells. One possibility may be to use peptides representing the Pax6 interaction surface. A promising study using a peptide that mimics the function of the short splice variant of the coregulator MTA1 is shown to sequester ERα in the cytoplam [44]. The MTA1s peptide had the ability to inhibit ERα-mediated transactivation, estrogen-dependent proliferation, anchorage-independent growth and *in vivo* tumor progression [45]. This indicates that development of peptides abrogating receptor-coactivator interactions may have therapeutical opportunities.

We found that Pax6 and SPBP colocalized with AR in the nucleus of androgen stimulated cells overexpressing the proteins. But since the expression of Pax6 and AR in various tissues is very restricted, few tissues coexpress them both. AR is highly expressed in the epithelium of the tear producing lachrymal glands surrounding the eyes [1], where also Pax6 is found. The expression patterns of *AR* and *PAX6* during development of the pituitary have a transient overlap along the midline border [46], [47]. Since the pituitary is the superior hormone-producing gland of the body, a possible common function for Pax6 and AR in this organ is to coparticipate in expression of hormones. Likewise, AR is coexpressed with Pax6 in distinct pyramidal neurons of the hippocampus [48], [49], in neurons and astrocytes of the developing and adult forebrain [50], [51], as well as in the dopamine-producing substantia nigra of the midbrain [52], [53]. Furthermore, our data show that Pax6 and AR are coexpressed in the androgen insensitive prostate cancer cell line PC3, the lens epithelial cell line B3, in addition to the cancer cell lines HEK293 and HeLa. Hence, the Pax6-mediated repression of AR activity may be important and relevant in certain tissues, and also in specific cancer cells aberrantly expressing both proteins.

In conclusion, SPBP and Pax6 modulate AR activity by using their DBDs to interact with the DBD of AR. Elucidating the specific mechanisms involved in the competition between Pax6 and SPBP for binding to AR, may result in important knowledge in the field of targeting AR coregulators for new cancer therapeutics.

## Supporting Information

Figure S1 **Expression levels of exogenous HA-AR, HA-SPBP and HA-Pax6 in HEK293 cells.** Subconfluent HEK293 cells in a 6 well dish (Nunc) were cotransfected with pDestHA-AR (375 ng), pDestHA-SPBP (1.5 µg), and pDestHA-Pax6 (250 ng), and stimulated with 10^−7^ M R1881. The cells were harvested in 2×SDS gel loading buffer approximately 20 hours after transfection, proteins separated by SDS-PAGE and visualized by Western Blotting using mouse anti-HA (1∶1.000, clone 12CA5, Roche) antibody. Stars indicate unspecific bands.
(TIF)

Figure S2 **The interaction between Pax6 and AR or SPBP and AR is not dependent on DNA.** GST, GST-AR(Central), and GST-AR(DBD) immobilized on glutathione sepharose beads were used to pull down *in vitro* translated ^35^S-labeled Pax6 (**A**) and SPBP (**B**) in the presence or absence of benzonase. The strength of the interactions is unaffected by removing DNA from the reactions.
(TIF)

Figure S3 **Members from all subgroups of the Pax family interact with the central region of AR.** GST and GST-AR(Central) immobilized on glutathione sepharose beads were used to pull down *in vitro* translated ^35^S-labeled members of the Pax family. The results show that all Pax proteins tested bind to the central region of AR.
(TIF)

Table S1 **Plasmids used in this study.**
(DOC)

Table S2 **Oligonucleotides used in this study.**
(DOC)
