# Supplementary figures and images for: Pax6 Represses Androgen Receptor-Mediated Transactivation by Inhibiting Recruitment of the Coactivator SPBP
Source: PLoS One. 2011 Sep 15;6(9):e24659. doi: 10.1371/journal.pone.0024659 (PMC3174178; doi:10.1371/journal.pone.0024659)

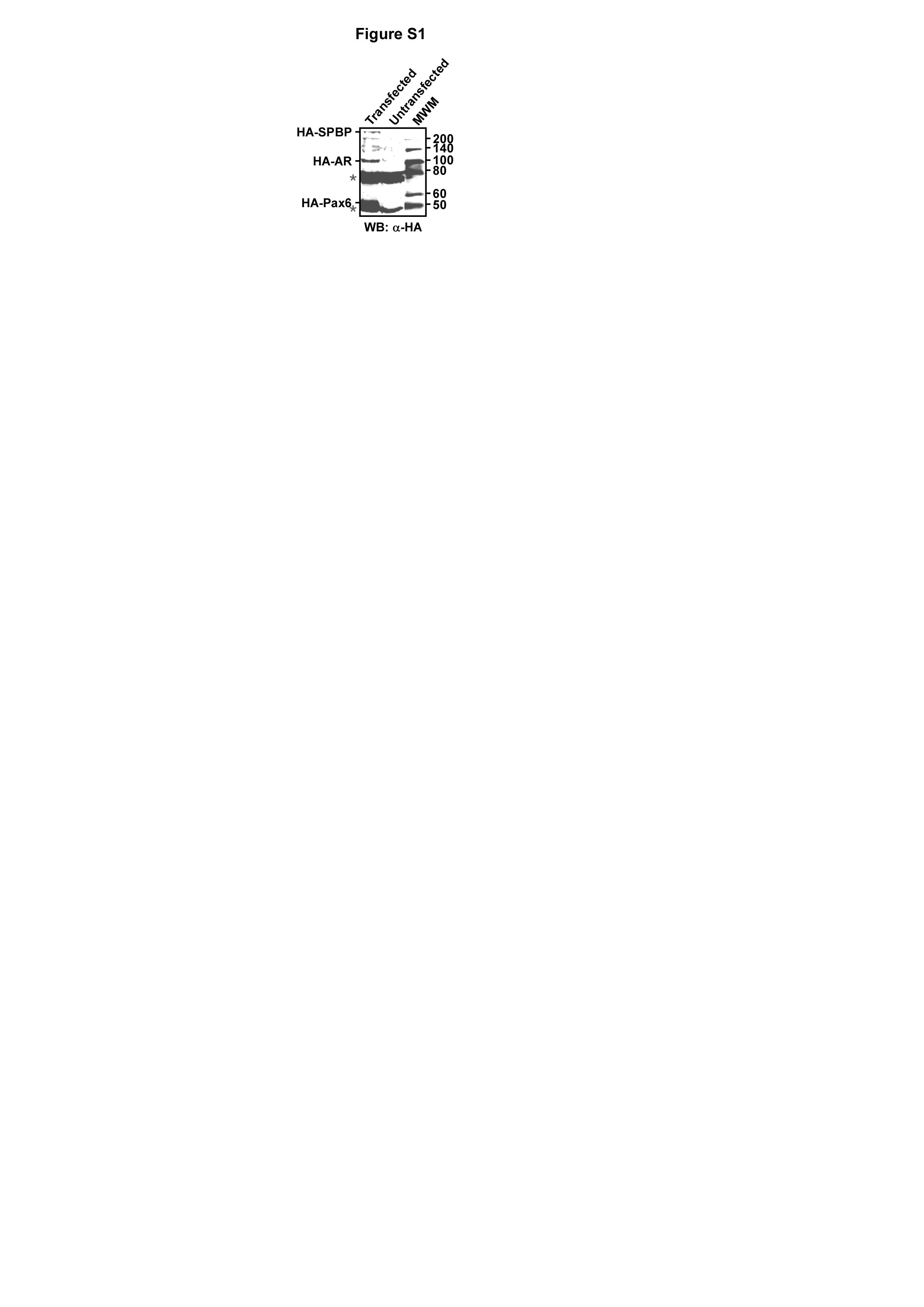

Supplement: Figure S1 — Expression levels of exogenous HA-AR, HA-SPBP and HA-Pax6 in HEK293 cells. Subconfluent HEK293 cells in a 6 well dish (Nunc) were cotransfected with pDestHA-AR (375 ng), pDestHA-SPBP (1.5 µg), and pDestHA-Pax6 (250 ng), and stimulated with 10−7 M R1881. The cells were harvested in 2×SDS gel loading buffer approximately 20 hours after transfection, proteins separated by SDS-PAGE and visualized by Western Blotting using mouse anti-HA (1∶1.000, clone 12CA5, Roche) antibody. Stars indicate unspecific bands. (TIF) [file pone.0024659.s001.tif]

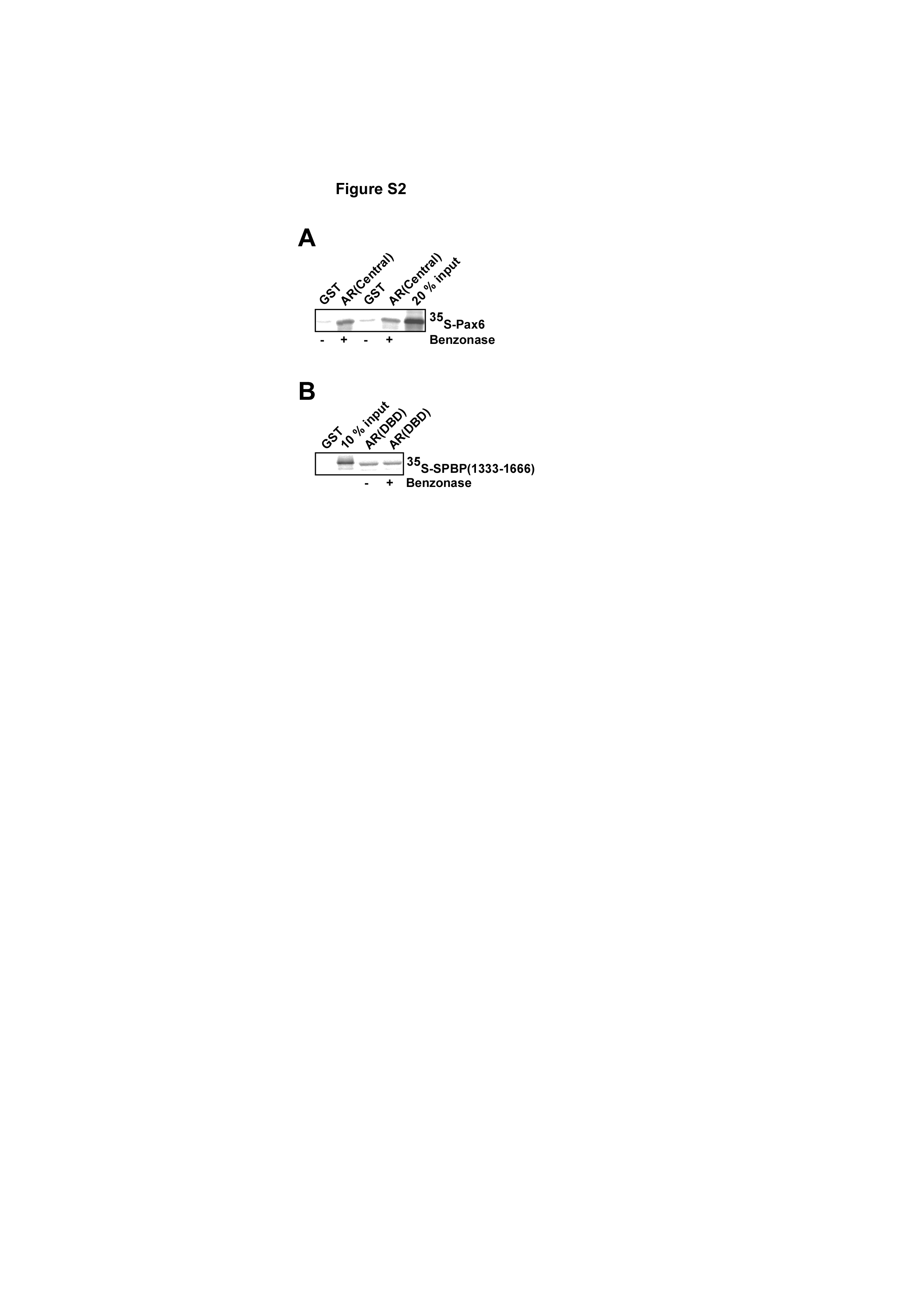

Supplement: Figure S2 — The interaction between Pax6 and AR or SPBP and AR is not dependent on DNA. GST, GST-AR(Central), and GST-AR(DBD) immobilized on glutathione sepharose beads were used to pull down in vitro translated 35S-labeled Pax6 (A) and SPBP (B) in the presence or absence of benzonase. The strength of the interactions is unaffected by removing DNA from the reactions. (TIF) [file pone.0024659.s002.tif]

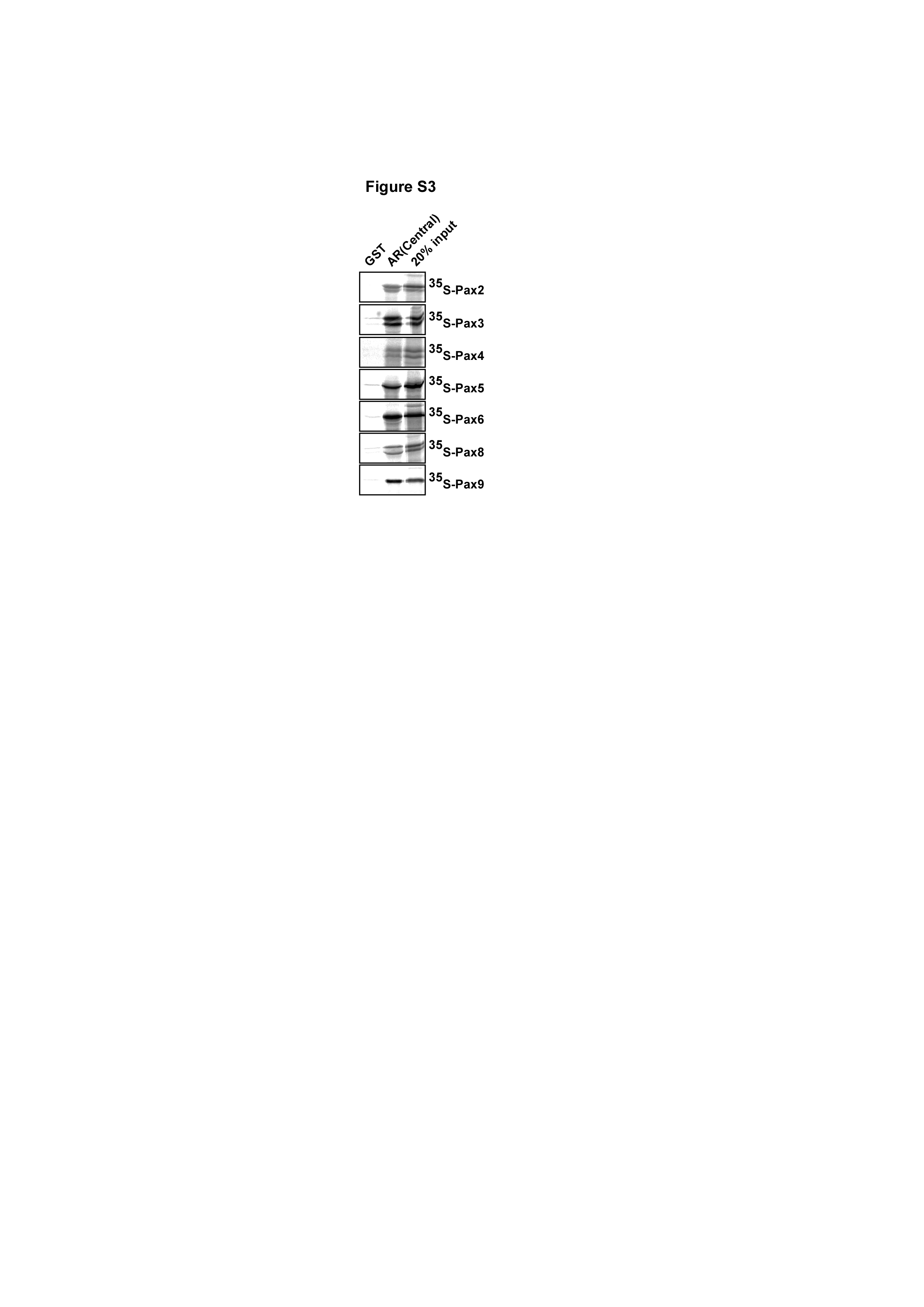

Supplement: Figure S3 — Members from all subgroups of the Pax family interact with the central region of AR. GST and GST-AR(Central) immobilized on glutathione sepharose beads were used to pull down in vitro translated 35S-labeled members of the Pax family. The results show that all Pax proteins tested bind to the central region of AR. (TIF) [file pone.0024659.s003.tif]
