# Supplementary material for: Pax6 Represses Androgen Receptor-Mediated Transactivation by Inhibiting Recruitment of the Coactivator SPBP
Source: PLoS One. 2011 Sep 15;6(9):e24659. doi: 10.1371/journal.pone.0024659 (PMC3174178; doi:10.1371/journal.pone.0024659)
Supplement: Table S1 — Plasmids used in this study. (DOC) [file pone.0024659.s004.doc]

**Table S1: Plasmids used in this study**

| **Vectors** | | **Description** | | | **Source** |
| --- | --- | --- | --- | --- | --- |
| ***Gateway cloning vectors*** | | | | | |
| pEntr1A | | Entry vector | | | Invitrogen |
| pEntr2B | | Entry vector | | | Invitrogen |
| pDonor207 | | Donor vector | | | Invitrogen |
| pEntr2B-re7 | | For gateway into pDestECFP-EYFP | | | This study |
| pDonor221 | | Donor vector | | | Invitrogen |
| pDest15 | | Bacterial GST fusion expression vector | | | Invitrogen |
| pDestTH1 | | Bacterial MBP fusion expression vector | | | [1] |
| pDestHA | | Mammalian HA fusion expression vector | | | [2] |
| pDest53 | | Mammalian GFP fusion expression vector; | | | Invitrogen |
| pDestEGFP-C1 | | Mammalian EGFP fusion expression vector | | | [2] |
| pDestmCherry-C1 | | Mammalian mCherry fusion expression vector | | | [3] |
| pDestEYFP-N1 | | Mammalian C-terminal EYFP fusion expression vector | | | [4] |
| pDestECFP-C1 | | Mammalian N-terminal ECFP fusion expression vector | | | [4] |
| pDestECFP-EYFP | | Mammalian expression vector for making fusion proteins with a double tag | | | T. Lamark |
| pDest3xFlag | | Mammalian triple Flag tag fusion expression vector | | | [5] |
| ***Other vectors*** | | | | | |
| pEGFP-C1 | | Mammalian EGFP fusion expression vector | | | Clontech |
| pGEX2T | | Bacterial GST expression vector | | | Amersham Biosciences |
| pcDNA3HA | |  | | | Invitrogen |
| pcDNA5-FRT/TO | |  | | | Invitrogen |
| ***Constructs made by traditional subcloning (this study)*** | | | | | |
| pEntr1A-AR | Full-length human AR PCR amplified from pSG5-AR | | | | |
| pEntr1A-AR(N-term) | Encoding amino acids 1-502 of AR | | | | |
| pEntr1A-AR(Central) | Encoding amino acids 503-680 of AR | | | | |
| pEntr1A-AR(C-term) | Encoding amino acids 680-919 of AR | | | | |
| pEntr2B-AR(N-DBD) | Encoding amino acids 500-550 of AR | | | | |
| pEntr2B-AR(DBD) | Encoding amino acids 550-640 of AR | | | | |
| pEntr2B-AR(Hinge) | Encoding amino acids 631-682 of AR | | | | |
| pDonor207-SPBP(532-987) | Amino acids 532-987 of SPBP subcloned from pEntr1A-SPBP | | | | |
| pDonor207-SPBP(989-1344) | Amino acids 989-1344 of SPBP subcloned from pEntr1A-SPBP | | | | |
| pDonor221-SPBP(1333-1960) | Amino acids 1333-1960 of SPBP subcloned from pEntr1A-SPBP | | | | |
| pcDNA5/FRT/TO-3xFlag-Pax6 | 3xFlag-Pax6 subcloned into pcDNA5FRT/TO from pDest3xFlag-mPax6 to make the HeLa FlpIn 3xFlag-Pax6 inducible cell line | | | | |
| ***Other constructs made by traditional subcloning*** | | | | | |
| pEntr1A-SPBP | Human SPBP | | | |  |
| pEntr1A-SPBP(ePHD) | ePHD of human SPBP | | | |  |
| pEntr1A-Pax6 | Mouse Pax6 | | | | [6] |
| pEntr1A-zfPax6 | Zebrafish Pax6 | | | | [7] |
| pcDNA3HA-Pax6 | Zebrafish Pax6 | | | | [8] |
| pcDNA3HA-Pax6ΔHD | Zebrafish Pax6 lacking the HD | | | | [8] |
| pcDNA3HA-Pax6ΔPD | Zebrafish Pax6 lacking the PD | | | | [8] |
| p285PB-Luc |  | | | | [9] |
| pCMV-βgal |  | | | | Stratagene |
| ***Constructs made by gateway LR reactions (this study)*** | | | | | |
| pDestEGFP-AR | | | pDestGST-AR(C-term) | pDestHA-SPBP(ePHD) | |
| pDestHA-AR | | | pDestMBP-AR(N-DBD) | pDestHA-SPBP(532-987) | |
| pDestECFP-AR | | | pDestMBP-AR(DBD) | pDestHA-SPBP(989-1344) | |
| pDestGST-AR(N-term) | | | pDestMBP-AR(Hinge) | pDestHA-SPBP(1333-1960) | |
| pDestGST-AR(Central) | | | pDestCherry-SPBP | pDest3xFlag-Pax6 | |
| pDest53-AR | | | pDestECFP-Pax6-EYFP | pDestECFP-re7-EYFP | |
| pDestHA-Pax6 | | |  |  | |
| ***Constructs made by gateway LR reactions*** | | | | | |
| pDestHA-SPBP | |  | | | [10] |
| pDestEGFP-SPBP | |  | | | [10] |
| pDestHA-SPBP(4-486) | |  | | | [10] |
| pDestHA-SPBP(1333-1666) | |  | | | [10] |
| pDestEGFP-Pax6 | |  | | | [6] |
| pDestCherry-Pax6 | |  | | | [6] |
| pDestGST-Pax6(HD) | |  | | | [8] |
| pDestGST-Pax6(PD) | |  | | | [7] |
| pDestPax6-EYFP | |  | | | [7] |
| pDestECFP-Pax6 | |  | | | [7] |

1. Hammarström M, Hellgren N, van den Berg S, Berglund H, Härd T (2002) Rapid screening for improved solubility of small human proteins produced as fusion proteins in Escherichia coli. Protein Sci, 11: 313-321.

2. Lamark T, Perander M, Outzen H, Kristiansen K, Øvervatn A, et al. (2003) Interaction Codes within the Family of Mammalian Phox and Bem1p Domain-containing Proteins. J Biol Chem, 278: 34568-34581.

3. Pankiv S, Clausen TH, Lamark T, Brech A, Bruun JA, et al. (2007) p62/SQSTM1 Binds Directly to Atg8/LC3 to Facilitate Degradation of Ubiquitinated Protein Aggregates by Autophagy. J Biol Chem, 282: 24131-24145.

4. Simpson JC, Wellenreuther R, Poustka A, Pepperkok R, Wiemann S (2000) Systematic subcellular localization of novel proteins identified by large-scale cDNA sequencing. EMBO Rep, 1: 287-292.

5. Jain A, Lamark T, Sjøttem E, Larsen KB, Awuh JA, et al. (2010) p62/SQSTM1 Is a Target Gene for Transcription Factor NRF2 and Creates a Positive Feedback Loop by Inducing Antioxidant Response Element-driven Gene Transcription J Biol Chem, 285: 22576-22591.

6. Elvenes J, Sjøttem E, Holm T, Bjørkøy G, Johansen T (2010) Pax6 localizes to chromatin-rich territories and displays a slow nuclear mobility altered by disease mutations. Cell Mol Life Sci, 67: 4079-4094.

7. Bruun JA, Thomassen EI, Kristiansen K, Tylden G, Holm T, et al. (2005) The third helix of the homeodomain of paired class homeodomain proteins acts as a recognition helix both for DNA and protein interactions. Nucleic Acids Res, 33: 2661-2675.

8. Mikkola I, Bruun JA, Holm T, Johansen T (2001) Superactivation of Pax6-mediated transactivation from paired domain-binding sites by dna-independent recruitment of different homeodomain proteins. J Biol Chem, 276: 4109-4118.

9. Palvimo JJ, Reinikainen P, Ikonen T, Kallio PJ, Moilanen A, et al. (1996) Mutual transcriptional interference between RelA and androgen receptor. J Biol Chem, 271: 24151-24156.

10. Sjottem E, Rekdal C, Svineng G, Johnsen SS, Klenow H, et al. (2007) The ePHD protein SPBP interacts with TopBP1 and together they co-operate to stimulate Ets1-mediated transcription. Nucleic Acids Res, 35: 6648-6662.
