# Supplementary material for: Pax6 Represses Androgen Receptor-Mediated Transactivation by Inhibiting Recruitment of the Coactivator SPBP
Source: PLoS One. 2011 Sep 15;6(9):e24659. doi: 10.1371/journal.pone.0024659 (PMC3174178; doi:10.1371/journal.pone.0024659)
Supplement: Table S2 — Oligonucleotides used in this study. (DOC) [file pone.0024659.s005.doc]

**Table S2: Oligonucleotides used in this study**

| 5´-hAndro.R | 5-GGCGGATCCTGGAAGTGCAGTTAGGGCTGGG |
| --- | --- |
| 3´-hAndro.R | 5-GGACTCGAGTCACTGGGTGTGGAAATAGATGGG |
| 5´-hAndro.R1 | 5-CAGGGATCCGGTACCCTGGCGGCATGGTGAG |
| 3´-hAndro.R1 | 5-GACCTCGAGCTCAATGGCTTCCAGGACATTCAG |
| 5´-hAndro.R2 | 5-CAGGGATCCCAGGTGTAGTGTGTGCTGGACAC |
| 3´-hAndro.R2 | 5-GACCTCGAGACACATCAGGTGCGGTGAAGTCG |
| 5´-hAndro.R3 | 5-GCAGGATCCCATTGACTATTACTTTCCACCCCAG |
| 3´-hAndro.R3 | 5-CAGCTCGAGTAGTTTCAGATTACCAAGTTTCTTCAG |
| 5´-hAndro.R5 | 5-GCAGGATCCTGATGTGTGGTACCCTGGCGGC |
| 3´-hAndro.R5 | 5-CCACTCGAGAATGGGCAAAACATGGTCCCTGGC |
| 5´-hAndro.R6 | 5-CGAGGATCCGCTGAAGAAACTTGGTAATCTGAAAC |
| 3´-hAndro.R6 | 5-GACCTCGAGCTCAATGGCTTCCAGGACATTCAG |
| 5´-SPBP(532-987) | 5-GGGGACAAGTTTGTACAAAAAAGCAGGCTGCGAGAGAGTGCGGCAAC |
| 3´-SPBP(532-987) | 5-GGGGACCACTTTGTACAAGAAAGCTGGGTTGGCGTGGGTCTGCTGTC |
| 5´-SPBP(989-1344) | 5-GGGGACAAGTTTGTACAAAAAAGCAGGCTATGCGGCGGGTCCCTGGCAG |
| 3´-SPBP(989-1344) | 5-GGGGACCACTTTGTACAAGAAAGCTGGGTGTGGGGGCAGTATTTTGGTCT  TAGCAGGG |
| 5´-SPBP(1333-1960) | 5-GGGACAAGTTTGTACAAAAAAGCAGGCTGCCCTGCTAAGACCAAAATAC  TGCC |
| 3´-SPBP(1333-1960) | 5-GGGGACCACTTTGTACAAGAAAGCTGGGTCCCCCCGCTCCGACTGCTCT  GT |
| 5´-3xFlag Pax6 | 5-GGCACAGGCCTACCATGGACTACAAAGACCATGACG |
| 3´-3xFlag Pax6 | 5-CGAGGACTCGAGGCTTTACTGTAATCGAGGCCAGTAC |
| 285PB-Luc-fw | 5-TAGGGACATAAAGCCCACAA |
| 285PB-Luc-rev | 5-TGATTGGCATGGGCAGTGTC |
| PSA-fw | 5-GGCAGCATTGAACCAGAG |
| PSA-rev | 5-TGAAGCACACCATTACAGAC |
| Actin-fw | 5-TGACGGTCAGGTCATCACTATCGGCAATGA |
| Actin-rev | 5-TTGATCTTCATGGTGATAGGAGCGAGGGCA |
